# A hybrid volumetric dose verification method for single‐isocenter multiple‐target cranial SRS

**DOI:** 10.1002/acm2.12430

**Published:** 2018-08-15

**Authors:** Saeed Ahmed, Jeff Kapatoes, Geoffrey Zhang, Eduardo G. Moros, Vladimir Feygelman

**Affiliations:** ^1^ Department of Radiation Oncology Moffitt Cancer Center Tampa FL USA; ^2^ Department of Physics University of South Florida Tampa FL USA; ^3^ Sun Nuclear Corp. Melbourne FL USA

**Keywords:** Patient‐specific SRS QA

## Abstract

A commercial semi‐empirical volumetric dose verification system (PerFraction [PF], Sun Nuclear Corp.) extracts multi‐leaf collimator positions from the electronic portal imaging device movies collected during a pre‐treatment run, while the rest of the delivered control point information is harvested from the accelerator log files. This combination is used to reconstruct dose on a patient CT dataset with a fast superposition/convolution algorithm. The method was validated for single‐isocenter multi‐target SRS VMAT treatments against absolute radiochromic film measurements in a cylindrical phantom. The targets ranged in size from 0.8 to 3.6 cm and in number from 3 to 10 per plan. A total of 17 films rotated at different angles around the cylinder axis were analyzed. Each of 27 total targets was intercepted by at least one film, and 2–4 different films were analyzed per plan. Film dose was always scaled to the ion chamber measurement in a high‐dose, low‐gradient area deliberately created at the isocenter. The planar dose agreement between PF and film using 3%(Global dose‐difference normalization)/1 mm gamma analysis was on average 99.2 ± 1.1%. The point dose difference in the low‐gradient area in the middle of every target was below 3%, while PF‐reconstructed and film dose centroids for individual targets showed submillimeter agreement when measured on a well aligned accelerator. Volumetrically, all voxels in all plans agreed between PF and the primary treatment planning system at the 3%/1 mm level. With proper understanding of its advantages and shortcomings, the tool can be applied to patient‐specific QA in routine radiosurgical clinical practice.

## 1 INTRODUCTION

Brain metastases are a common oncological diagnosis^1^ and intracranial stereotactic radiosurgery (SRS) has evolved as an important modality of treatment/palliation for that disease.^2^, ^3^ It was demonstrated that even with multiple metastases the SRS treatment could provide reasonable local control,^4^, ^5^ and a multi‐institutional observational study suggested that clinical outcomes for patients with 5–10 individual metastases treated by SRS alone may be non‐inferior to those with 2–4 targets.^6^, ^7^ While conceptually straightforward in principle, multi‐target SRS poses logistical challenges. As the number of treated metastases increases, the traditional SRS paradigm of one isocenter per lesion leads to prohibitively long treatment times and had to be revisited with the goal of simultaneously treating multiple targets. Interestingly, while dynamic conformal arcs were the mainstay of linac‐based radiosurgery for years, the feasibility of single‐isocenter multiple‐target (SIMT) approach was first demonstrated with a relatively new volumetric modulated arc therapy (VMAT) technique.^8^, ^9^ The most recent commercial implementation (HyperArc, Varian Medical Systems) offers refinements in terms of planning automation and collision prevention.^10^ Alternatively, Huang et al.^11^ proposed a concept of single isocenter dynamic conformal arcs (SIDCA), whereby each lesion is treated by a dedicated group of dynamic conformal arcs but all groups share the same isocenter positioned between all targets. This allows for more efficient dynamic arc treatment, as only one isocenter setup is necessary, and the couch angles and arc directions can be optimized for fastest delivery. A version of SIDCA is commercially implemented in Automatic Brain Metastases Planning Element software by BrainLab.^12^, ^13^ It creates a series of dynamic arcs and each lesion can be covered by all or some of them, depending on the relative position, to minimize normal tissue irradiation. Both techniques by necessity produce treatment plans containing complex MLC apertures, and it is prudent to perform patient‐specific end‐to‐end test prior to commencing the treatment.^14^ The number of small, off‐center targets poses a unique challenge to dosimetry devices commonly used for such tests. The approach should possess high spatial resolution as the lesions could be of the order of 1 cm or less in size. At the same time, the targets could be fairly wide spread, which negates the advantages of dedicated “stereotactic” detector arrays with small detector pitch, that typically have a relatively small active area under the assumption that the lesion would be located at isocenter.^15^ Moreover, the targets randomly placed in three dimensions naturally call for a 3D verification approach. The only true 3D dosimeters with high spatial resolution are radiochromic gels/polymers,^16^, ^17^ one of which was successfully used for VMAT‐based SIMT validation.^18^ However, volumetric radiochromic dosimetry is sufficiently cumbersome at this point to prevent its use for routine patient‐specific quality assurance.^19^ Therefore, a more practical method is needed that combines some high spatial resolution measurements with 3D dose reconstruction over a volume of an adult head. One such approach, which we validate in this paper, is a hybrid technique whereby information collected from the accelerator electronic portal imaging device (EPID) and delivery log files is supplied as input to the independent dose calculation algorithm that reconstructs the expected deliverable dose distribution on the patient CT dataset.^20^

## 2 METHODS

### 2.A System description

The method evaluated in this paper is a part of PerFRACTION (PF) software suite (Sun Nuclear Corp, Melbourne, FL) that provides a number of options for pre‐ and on‐treatment patient‐specific dosimetric analysis. We focused on the pre‐treatment patient‐specific QA (called Fraction 0) and chose the input configuration that, in our opinion, provided the most advantageous balance between the empirical and calculation portions of the analysis. The software runs on a central dedicated Windows server and all routine user interactions occur through a web browser‐based interface. At the heart of the method is the graphics processing unit‐accelerated superposition/convolution dose calculation algorithm described and validated previously.^21^, ^22^ The beam model can be customized by the vendor to fit the user's data, although a generic model for the accelerator class configuration proved sufficient in this work.

The system is compatible with contemporary Varian and Elekta linacs. The verification process starts with transferring the patient CT and finalized Plan, Structure, and Dose DICOM RT objects from the treatment planning system to PF. This establishes a new patient/plan in the system. The same plan is transferred to the record‐and‐verify (R&V) system and is then delivered to the EPID operating in a cine mode. The compressed (MPEG) EPID movies, one per beam, are stored after the delivery in a specified network directory that is monitored by PF, automatically transferred to the PF server, and associated with the individual beam(s) found in the RT Plan object. The accelerator log files are processed in the exact same fashion. The EPID image frames are then synchronized to the log files to determine the exact duration of time when each EPID frame was acquired. This is achieved by first creating a series of predicted images based on the projection of the RT Plan fluence to the plane of the EPID. The predicted images of every segment (or multitude of segments) are then compared to the measured frames to find the maximum similarity. The measured frame with maximum similarity is considered to be acquired during the same segments as the best matching predicted image.

With the synchronization process completed, the frames are then analyzed to determine the location of each MLC during that time period. An edge detection algorithm is used to find the MLC edges on EPID frames. From this information, an internal RT Plan is devised, with the control points (CP) created by amalgamation of the EPID movies and log files. Specifically, the time‐resolved multi‐leaf collimator (MLC) apertures are derived from the EPID files, independent of the accelerator logs. The positions of the rest of the accelerator axes per CP (fractional monitor units (MU), gantry angle, etc.) are determined from the delivery log files. In addition to MLC positions, if radiation is detected on regions of the EPID which were supposed to be covered by the jaws, corrected jaw positions are incorporated into the internal RT Plan so that such unexpected radiation is properly accounted for in the final dose calculation. With CP point information thus complete, the dose calculation is triggered. The PF calculation voxel size is the larger of the TPS or the minimum set in PF, which was 2.5 mm in this work. This voxel size was set to obtain a reasonable compromise between the calculation speed and accuracy and the dose distribution is not distinguishable from the one calculated with a 2 mm voxel^14^ at the 1% dose‐difference/1 mm distance to agreement level. The resulting semi‐empirical dose distribution can be compared to the planned one by standard gamma analysis^23^ and dose‐volume histogram evaluation.

### 2.B Planning and delivery

#### 2.B.1 The phantom

**Figure 1:**
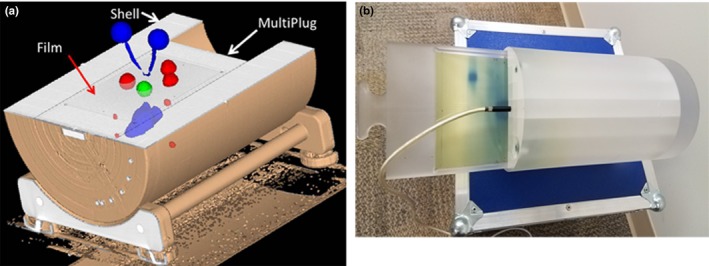
(a) A CT‐based coronal plane cut through the center of the assembled phantom. The inner cylinder, the outer shell and the film rectangle in the middle (coronal orientation) can all be appreciated. An example of ROI arrangement is presented, with multiple targets (red), normal structures (blue) and a central 2 cm target sphere (green) for ion chamber normalization, the latter common to all plans. (b) A photograph of the MultiPlug with the partially inserted film holder and ion chamber. Note that for the actual measurements the ion chamber insert replaced film at the isocenter.

A MultiPlug (Sun Nuclear) phantom is a 15.1 cm diameter Poly(methyl methacrylate) (PMMA) cylinder, further encased in a PMMA shell with 26.6 cm outer diameter (Fig. 1). The phantom has interchangeable inserts to accommodate either an ion chamber (in this case, 0.06 cm^3^ A1SL, Standard Imaging, Middleton, WI) or a 13.2 × 16.5 cm^2^ piece of radiochromic film. The film insert has small sharp points at five locations to imprint fiducial marks on the film. Those were augmented by small amounts of Barium paste to provide high‐contrast but low‐artifact fiducials for eventual cone‐beam CT (CBCT) alignment on a linear accelerator. The plug with the film insert can be freely rotated in the shell around the cylinder axis. The phantom was scanned on a 16‐slice Big Bore scanner (Philips Medical, Cleveland, OH) according to our standard SRS protocol (sequential scans with 1.25 mm slice thickness), with four different film plane orientations: coronal, sagittal, and ±45° obliques. The plane angular positions were established directly with a digital level. These scans served as the baseline datasets for CBCT alignment of the phantom with the film holder in different orientations.

#### 2.B.2 Treatment planning

The datasets were transferred to the TPS (Pinnacle v. 14.0, Philips Radiation Oncology Systems, Fitchburg, WI) and the isocenter was placed based on the known locations of the film fiducials visible on CT scans. The next step was devising regions of interest (ROI) for planning. Six plans of two types were created. The first three plans in Table 1 contain only three spherical target ROIs each, with the goal of creating conformal plans without additional constraints. Each target is intersected in the middle by at least one film plane. Plans 4–6 are rooted in real patient datasets. The patient RT Structure DICOM objects were processed to make transfer to the phantom CT possible. The organs‐at‐risk (OAR) had to be moved some to ensure that they were positioned within the cylinder. The targets were also nudged to intersect with at least one film plane each. An example arrangement can be seen in Fig. 1A, which shows the targets (red) above, below, and intersecting the coronal plane. Two to four planes were measured per plan. Overall, 17 films intersecting 27 targets were analyzed. In addition to those, each plan contained a 2 cm diameter spherical structure (green in Fig. 1A) drawn at the isocenter and planned to achieve uniform 18 Gy dose for normalization purposes.

**Table 1:** The plans, target sizes and positions, and film plane orientations. The maximum filed sizes (jaws) for each plan are also presented

Plan	No. of Targets	Targets max dimensions, cm	Target center distance from isocenter, cm	Max. field size (X × Y), cm^2^	Measurement planes orientation
1	3	1.3, 1.2, 2.1	3.6, 5.0, 5.5	10.1 × 12.0	Cor.,Obl.45°,Obl.135°
2	3	1.1, 1.2, 2.4	5.8, 5.9, 6.2	12.7 × 15.0	Cor.,Sag.,Obl.135°
3	3	1.3, 1.2, 2.9	5.5, 4.5, 4.7	11.1 × 13.0	Obl.45°,Obl.135°
4	3	2.2, 1.1, 0.8	4.3, 3.6, 5.4	11.3 × 10.5	Obl.45°,Obl.135°
5	5	2.2, 1.1, 0.8, 3.6, 2.3	4.3, 3.6, 5.4, 4.7, 4.0	10.9 × 12.5	Cor.,Obl.45°,Ob1.135°
6	10	2.2, 1.1, 0.8, 3.6, 2.3, 1.4, 1.4, 0.9, 1.2, 1.1	4.3, 3.6, 5.4, 4.7, 4.0, 4.0, 5.1, 6.3, 3.4, 6.3	12.2 × 12.5	Cor.,Sag.,Obl.45°, Obl.135°

VMAT optimization employed two full coplanar and two partial (164° rotation) non‐coplanar (±25° table rotation) arcs. The flat caudal edge of the phantom precluded the use of vertex beams common in SRS, which however should not affect the generality of the tests. All plans used A 6 MV flattening filter free beam with the maximum repetition rate of 1400 MU/min and were calculated with 2° CP increment and a 2 mm isotropic dose grid resolution. The prescriptions followed RTOG 0320 protocol,^24^ depending on the target size: 24 Gy to the planning target volume (PTV) <2 cm, 18 Gy to the PTV between 2.1–3 cm, and 15 Gy to the 3.1–4 cm PTV. Plans 4‐6 also employed common OAR objectives from the same protocol.

#### 2.B.3 Beam delivery

All experiments were performed on a TrueBeam v.2.5 linear accelerator (Varian Medical Systems, Palo Alto, CA) equipped with a standard 120‐leaf Millennium MLC, a 6 degree of freedom (6DOF) couch, and an aS1000 EPID. The EPID pixel size is 0.39 mm, which translates into 0.26 mm effective size at isocenter when the EPID is positioned 150 cm from the source. Prior to measurements the alignment of mechanical, MV, and kV isocenters of the accelerator were verified by two methods. First, the built‐in IsoCal verification routine^25^ was employed. It demonstrated the maximum MV isocenter deviation of 0.42 mm (with no couch rotation) and negligible translational and angular misalignment of the kV and MV imagers. Second, an independent MLC‐based Winston‐Lutz test with 12 angular combinations covering the full range of accelerator motions confirmed the maximum treatment isocenter deviation of 0.41 mm.

The plan information was transferred to the accelerator through Mosaiq v. 2.4 (Elekta Impac, Sunnyvale, CA) R&V system. Before delivery, the phantom was first leveled and then aligned in 3D by CBCT to the film fiducials in the desired plane orientation with the help of the 6DOF couch.

### 2.C Dose comparison

The core of this work is dosimetric comparisons between PF reconstructed dose and film measurements. The strategy was to convert the relative dose measured by calibrated film to absolute by normalization to the ion chamber dose at isocenter. To that end, the ion chamber reading in the MultiPlug phantom was collected under the standard conditions (parallel‐opposed horizontal 10 × 10 cm^2^ fields) and converted to dose by comparison with Pinnacle point dose in the same geometry. Subsequently, an ion chamber measurement was performed for each plan and the resulting dose at isocenter was used to scale dose for the films belonging to that plan.

#### 2.C.1 Film measurements

Extended range Gafchromic film (EBT‐XD, Ashland Inc., Bridgewater, NJ) was chosen because of the wide dynamic range,^26^, ^27^ well‐suited for SRS verification. An additional benefit is greatly reduced scanner lateral response artifact.^26^ Both calibration and measurement film pieces were scanned in the same orientation with respect to the original sheet they came from. Templates sized to fit the calibration (smaller) and measurement films were made out of black paper to reproducibly position the films in the center of the flatbed document scanner (Expression 11000XL, Epson Seiko Corporation, Nagano, Japan). Exposed films were scanned 24 h after irradiation, in transmission mode and without any corrections. Resolution was set at 72 dpi (0.35 mm/pixel). Every film was scanned in the same position and orientation before the exposure to account for any background non‐uniformity. Film calibration was performed with a 6MV beam in a water‐equivalent solid phantom in the dose range from 2 to30 Gy.

The films were analyzed with RIT v.6.6 software (Radiologic Imaging Technology, Inc., Colorado Springs, CO). The film scans were transferred to RIT as 48 bit color image files. The background correction was applied using the built‐in routine. The film fiducials marks were aligned to the pre‐defined geometric template, and the dose after application of the calibration curve was further scaled to match the ion chamber dose at the isocenter. The film dose was averaged for scaling over 13 central pixels in the craniocaudal direction, corresponding approximately to the chamber active volume length. There is no direct interface for importing dose in arbitrary plane from PF to RIT. Instead, volumetric dose was exported from PF as a DICOM RT Dose file and then imported to Pinnacle using a custom script. After that, planar doses in required orientations could be extracted from Pinnacle on a 1 mm grid using the built‐in IMRT QA tool and imported into RIT. Three types of tests were performed using RIT. First, an overall dose comparison was done using gamma analysis with 3% (global dose‐difference normalization), 1 mm distance to agreement, and 10% low‐dose cutoff threshold criteria. The RIT digital gamma analysis routine modeled after Depuydt et al^28^ was used. For completeness, the same analysis was performed for Pinnacle. Second, the point doses in the low‐gradient region near the center of each target were extracted from the film dose profiles (averaged over 3 pixels, or about 1 mm) and compared to PF. The distribution of dose‐differences was tested for normality by D'Agostino & Pearson test implemented in GraphPad Prism statistical package (v. 7.0, GraphPad Software, La Jolla, CA). Finally, paramount to radiosurgical applications, the alignment of measured and reconstructed profiles at the 50% level (dose centroid) was evaluated. Horizontal and vertical profiles were drawn in RIT through the center of each target on every film image. The vertical profile always corresponded to the craniocaudal direction. The horizontal profile anatomical direction varied with film orientation, anywhere from anteroposterior to lateral, and the results were segregated accordingly. The metric was, in most cases, the difference in the coordinates of the midpoints between the 50% level dose profile points. In a few instances where the targets were too close to each other to produce clearly isolated dose peaks on the film, the 65% profile points were used to calculate the dose centroid.

## 3 RESULTS

### 3.A Gamma analysis results

The gamma analysis results (3%G/1 mm) are detailed in Table 2. Excellent agreement is observed for PF, with the lowest passing rate of 96.1%. Pinnacle results are also solid, although for two films the passing rate slipped just below 90%. The 95% confidence intervals were 98.7–99.8% for PF and 95.2–98.4% for Pinnacle, indicating that both systems can be considered in agreement with experiment by current standards.^29^ It is therefore not surprising that volumetric gamma analysis comparison between the two algorithms demonstrated 100% agreement for all plans at the 3%/1 mm level.

**Table 2:** Planar gamma analysis passing rates (3%G/1 mm) for PF and Pinnacle vs. film

Plan	No. of Targets	Planes	Gamma passing rate (%)	
			PF vs. Film	Pinnacle vs. film
			100	97.7
		Obl.45°	96.1	89.4
		Obl.135°	99.1	99.0
2	3	Cor.	100	98.1
		Sag.	99.4	95.7
		Obl.135°	99.8	99.3
3	3	Obl.45°	100	98.3
		Obl.135°	99.9	99.2
4	3	Obl.45°	100	97.1
		Obl.135°	100	99.4
5	5	Cor.	99.4	89.9
		Obl.45°	99.7	98.6
		Obl.135°	97.3	94.5
6	10	Cor.	98.3	94.3
		Sag.	99.6	99.0
		Obl.45°	99.3	97.5
		Obl.135°	99.4	98.4
		Ave	99.2	96.8
		SD	1.1	3.1

**Figure 2:**
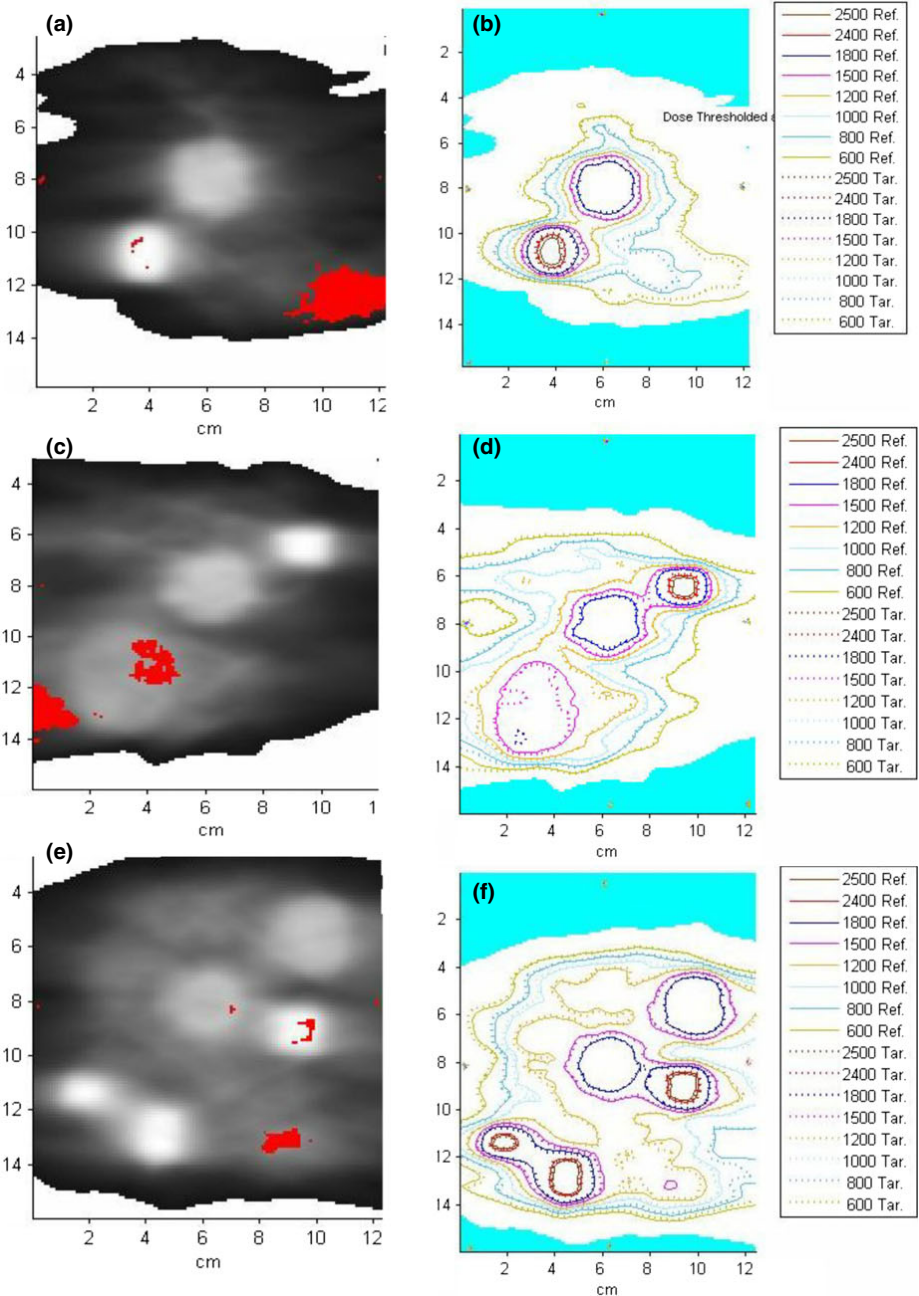
Gamma maps and isodose overlays for PF vs. film. (a&b): Oblique 45° plane from Plan 1, (c&d): Oblique 135° plane from Plan 5, and (e&f): Oblique 45° plane from Plan 10.

Fig. 2 illustrates the patterns of gamma analysis failures for the films with the lower passing rates. While some minor discrepancies in the target areas are present, the majority of the disagreement for both PF and Pinnacle, which is already quite small in absolute terms, is confined to the low‐ or intermediate‐dose regions. The latter are sometimes prominent when a film plane glances the target and some of the peripheral target dose is still evident on the film. In that case, there is a high‐dose gradient in the direction perpendicular to the film, leading to dosimetric discrepancies due to residual geometric misalignments. Those errors are not accounted for in the distance‐to‐agreement since the gamma analysis is performed in two dimensions (the film plane).

### 3.B Peak target dose

Both PF and Pinnacle show agreement with measurement largely to within ±3%, which is satisfactory, particularly for the targets less than 1 cm in size. For all targets, the average dose‐errors were −0.4% ± 1.3% (range −2.2 to 2.4%, 99% CI −1.1 to 0.3%) and 0.1% ± 1.6% (range −2.7 to 3.2%, 99% CI −0.8 to 1.0%) for PF and Pinnacle respectively. Both distributions did not show significant deviation from normal by the D'Agostino & Pearson normality test (*P* ≥0.2). There was no correlation between the dose‐error and the target size (Pearson correlation coefficient *r* = 0.23 and 0.21 for PF and Pinnacle, respectively). Similarly, there was no correlation between the dose‐error and the target distance from isocenter (*r =* 0.1 and −0.1 for PF and Pinnacle, respectively).

### 3.C Profile alignment

The results of dose profiles alignment between PF and film are presented in Table 3. Within the range of accelerator motions employed in the plans, submillimeter average displacements between the reconstructed and planned dose distribution centroids can be inferred.

**Table 3:** Displacement between PF and film dose profiles centers in different anatomical directions

Direction	Craniocaudal	Anteroposterior	Lt‐Rt	Obl. 45°	Obl. 135°
No. analyzed	27	4	3	11	7
Δ ±1SD (mm)	−0.3 ± 0.4	0.0 ± 0.6	−0.1 ± 0.4	−0.2 ± 0.7	0.0 ± 0.4

## 4 DISCUSSION

While the recent AAPM TG‐218 report^29^ prescribes the error thresholds and action levels for gamma analysis comparison between measured and planned dose distributions, there is no such clear guidance for purely calculational or semi‐empirical verification. We chose to retain the 3% dose‐error threshold from TG‐218, which is also similar to the point‐dose verification recommendations for complex non‐IMRT beams.^30^ Given the tight SRS spatial accuracy expectations, a 1 mm distance‐to‐agreement threshold seemed desirable. Finally, the TG‐218 report unequivocally justifies global dose‐error normalization for routine patient‐specific QA. With these criteria, the system in question — PerFRACTION — was able to achieve on average 99.2 ± 1.1% agreement rates with absolute film measurements. Volumetrically, all voxels in all plans agreed between PF and the TPS at the same 3%/1 mm level. The point doses near the target center agreed between PF and film to better than 3%, for the target sizes ranging from 0.8 to 3.6 cm. The reconstructed dose centroid positions derived from the EPID‐measured MLC apertures on a well‐aligned accelerator showed on average sub‐millimeter displacements from film measurements. The studied system is thus sufficiently accurate in the radiosurgical setting for routine semi‐empirical dose reconstruction.

However, a bigger question remains on the role of calculations vs. measurements in patient‐specific dosimetric QA. It is a subject of ongoing debate,^31^ with the latest TG‐218 report^29^ acknowledging but not adjudicating the issue. We characterize the approach described in this paper as semi‐empirical or hybrid, as some of the information (MLC apertures) is derived from independent measurements, while other elements are harvested from the accelerator log files. In addition to the delivered MLC leaf positons, such approach definitively tests the integrity of the data transfer chain all the way from the TPS to the accelerator, which is one of the most important aspects of the patient‐specific end‐to‐end tests. The beam model quality, which is the frequent culprit in the end‐to‐end head and neck phantom irradiation failures^32^ is also tested, but not by direct dose measurements. We would argue that this level of scrutiny is acceptable for routine QA (as opposed to system commissioning), and furthermore 3D reconstruction with small voxels is more comprehensive than, for example, experimental planar sampling with large detector pitch arrays. Studying the sensitivity of the method to induced MLC errors is outside the scope of this work, but it is likely to be similar to the results demonstrated by others in the related work with high‐resolution systems.^33^, ^34^ While the risk of a false positive findings (an error reported when there is none) is easily mitigated by measurement if necessary, a potential false negative (no error reported when there is one) is more likely to slip through. The risk of accelerator absolute calibration changes after the morning checkout has always been considered sufficiently mitigated in conventional treatments by the redundancy of the dosimetry chain, and the TG‐218 specifically recommends the IMRT QA measured dose to be normalized to the daily output^29^ to exclude the influence of the fluctuations, which could otherwise consume a substantial part of the error budget. Thus, the remaining weakest link in the tested PF configuration (and there are other options, not described in this work) is the lack of independent verification of the accelerator axes positions (fractional MU, gantry angle, etc.) other than the MLC. If an accelerator fails in such a fashion that the log files reflect the intended plan that diverges from the delivered treatment, a dosimetric error may go unnoticed. It should be, however, argued that a random accelerator failure during treatment by definition cannot be reliably caught by *pre‐treatment* measurements in the first place, while any gradual parameter drift is more appropriately addressed by an ongoing comprehensive QA program. Regarding systematic delivery deficiencies, with modern digital accelerators, the known issues such as the overshoot phenomenon^35^ are largely considered mitigated.^20^ For example, for the TrueBeam accelerator with its 20 ms controller interrogation cycle and strict delivery linearity enforcement inside each control point,^36^ even the gantry acceleration trajectory is highly predictable and reproducible.^37^ Therefore, while not going as far as endorsing the log file analysis as a “premier SRS/SBRT QA tool,”^38^ we nevertheless suggest that coupled with thorough TPS commissioning and comprehensive ongoing accelerator QA program, the hybrid verification method validated in this paper is a viable tool that could be applied in clinical practice.

## 5 CONCLUSIONS

A semi‐empirical volumetric dose verification system extracts MLC positions from the EPID movies, while the rest of the delivery control point information comes from the accelerator log files. This combination is used to reconstruct dose on a patient CT dataset with a fast superposition/convolution algorithm. The method was comprehensively validated for single‐isocenter multi‐target VMAT SRS treatments against absolute film measurements. With proper understanding of its advantages and shortcomings, the tool can be used in routine clinical practice.

## CONFLICT OF INTEREST

SA is a graduate student supported by an SNC grant and VF is the PI on the project. JK is an SNC employee.
